# Editorial: Somatic genomic mosaicism & human disease

**DOI:** 10.3389/fgene.2022.1045559

**Published:** 2022-10-07

**Authors:** Ivan Y. Iourov, Henry H. Heng

**Affiliations:** ^1^ Yurov’s Laboratory of Molecular Genetics and Cytogenomics of the Brain, Mental Health Research Center, Moscow, Russia; ^2^ Vorsanova’s Laboratory of Molecular Cytogenetics of Neuropsychiatric Diseases, Veltischev Research and Clinical Institute for Pediatrics and Pediatric Surgery of the Pirogov Russian National Research Medical University of the Russian Ministry of Health, Moscow, Russia; ^3^ Department of Medical Biological Disciplines, Belgorod State University, Belgorod, Russia; ^4^ Center for Molecular Medicine and Genetics, Wayne State University School of Medicine, Detroit, United States; ^5^ Department of Pathology, Wayne State University School of Medicine, Detroit, United States

**Keywords:** somatic mosaicism, aging, brain disease, cancer, Alzheimer’s disease, chromosome, fuzzy inheritance, karyotype coding

Somatic genomic mosaicism has become a major focus of genetic research during the last decade (Campbell et al., 2015; D'Gama and Walsh, 2018). Considering the number of cellular divisions required to produce ∼10^14^ of cells in an average human being, it is highly unlikely that all these cells share identical genomes. Thus, all humans are apparently genetic mosaics (Iourov et al., 2012). This viewpoint is endorsed by the new genomic concept of “Fuzzy Inheritance” (Heng, 2019). With the introduction of new genomic technologies, somatic mosaicism has been found to be a mechanism for human morbidity. Additionally, somatic (chromosomal and single-gene) mosaicism appears to be a mechanism for human interindividual diversity, development and aging (Campbell et al., 2015; D'Gama and Walsh, 2018; Iourov et al., 2012; Heng, 2019; Vijg, 2014). More precisely, monogenic and chromosomal diseases, neurodevelopmental/neurobehavioral and neuropsychiatric disorders, neurodegeneration, cancer and healthy/unhealthy aging are associated with a wide spectrum of somatic genomic mosaicism types (D'Gama and Walsh, 2018; Iourov et al., 2012; Heng, 2019; Vijg, 2014; Iourov et al., 2019; Yurov et al., 2019; Vorsanova et al., 2020; Ye et al., 2020; Miller et al., 2021; Iourov et al., 2021a; Iourov et al., 2021b). According to the Genome Architecture Theory, somatic mosaicism-mediated heterogeneity is essential for cellular adaptation, and at the same time, as an evolutionary trade-off, somatic mosaicism may be a disease mechanism, as well (Heng, 2019; Ye et al., 2019; Iourov et al., 2020; Iourov et al., 2021b; Heng and Heng, 2021). Timely recognition of the importance of somatic mosaicism is required to understand genetic mechanisms of human morbidity and physiological changes during the ontogeny for improving life quality and span.

This Research Topic presents the knowledge about somatic genomic mosaicism acquired by molecular genetic and cytogenetic/cytogenomic studies. Moreover, original hypotheses about the role of somatic mosaicism in human diseases and innovative approaches to the detection are described.

The role of chromosome instability and mosaic aneuploidy in the pathogenesis of neurodegenerative and neurodevelopmental disorders is generally overlooked. The paper by Potter et al. fills this gap in current biomedical literature and describes a hypothesis suggesting the involvement of aneuploidy in the cognitive deficits that characterize the neurological symptoms of these disorders by promoting apoptosis in the diseased brain. The analysis of somatic chromosomal mosaicism is continued by Liehr and Al-Rikabi, who provided a timely systematic review of mosaic small supernumerary marker chromosomes detected in unaffected individuals.

Since Alzheimer’s disease is repeatedly associated with a variety of types of somatic mosaicism (Iourov et al., 2012; Yurov et al., 2019; Ye et al., 2020; Miller et al., 2021), it is not surprising that this common and devastative disease is a focus of four articles of this Research Topic. The description of somatic mosaicism in Alzheimer’s disease is started by a review by Bajic et al., who described the role of X chromosome-specific mosaicism and instability in the pathogenesis. Barrio-Alonso et al. hypothesize that neuronal hyperploidization is a highly probable mechanism of Alzheimer’s disease. Ueberham and Arendt review genomic indexing by somatic gene recombination of mRNA/ncRNA and suggest that related processes underlie several symptoms of Alzheimer’s disease. Still, this process probably has both advantageous and deleterious consequences. Finally, Alzheimer’s disease-associated somatic mosaicism is addressed by Kaeser and Chun. The authors present their original potentially unifying hypothesis suggesting that mosaic somatic gene recombination is a novel mechanism to explain the pathogenesis of this currently untreatable disease.

Recently, somatic mosaicism has been found to be involved in cancer pathogenesis (Iourov et al., 2021a; Iourov et al., 2021b; Heng and Heng, 2021). This involvement is highlighted by Ye et al., who used multiple myeloma as a model for describing cancerous aspects of somatic genomic mosaicism.

The Research Topic is finalized by two articles describing approaches to study somatic mosaicism. Kuroki et al. present a study performed for establishing quantitative PCR assays for active Long Interspersed Nuclear Element-1 (LINE1) subfamilies, which may be applied to the analysis of aging-associated retrotransposition, which is a common cause of somatic mosaicism. Dong et al. describe their original and freely available software tool (SCCNV), which may be used for identifying mosaic copy number variation by analysing single-cell whole-genome sequencing data.

Recently, a series of publications have further highlighted the importance of somatic chromosomal mosaicism in cancer and aging. Because karyotype codes the “system information” that organizes gene interactive networks, altered karyotypes represent newly formed information packages. The somatic chromosomal mosaicism should certainly alter genetic-environmental interactions offering therapeutic opportunities in disease and pathological aging. We regret that some of these papers are not included in this Research Topic, but readers are able to read them elsewhere (Ye et al., 2019; Iourov et al., 2020; Vorsanova et al., 2020; Ye et al., 2020; Iourov et al., 2021a; Iourov et al., 2021b; Heng and Heng, 2021; Miller et al., 2021) for complementing their views on somatic genomic mosaicism in humans.

To this end, we have to inform the readers that our co-editor, Svetlana G, Vorsanova, has tragically passed away during the finalization of this topic (for more information, please see (Iourov, 2022)). Accordingly, we dedicate our editorial and Research Topic to her memory.

## References

[B1] Campbell I. M. Shaw C. A. Stankiewicz P. Lupski J. R. (2015). Somatic mosaicism: Implications for disease and transmission genetics. Trends Genet. 31 (7), 382–392. 10.1016/j.tig.2015.03.013 25910407 PMC4490042

[B2] D'Gama A. M. Walsh C. A. (2018). Somatic mosaicism and neurodevelopmental disease. Nat. Neurosci. 21 (11), 1504–1514. 10.1038/s41593-018-0257-3 30349109

[B3] Heng H. H. (2019). Genome chaos: Rethinking genetics, evolution, and molecular medicine. Cambridge, MA, USA: Academic Press Elsevier.

[B4] Heng J. Heng H. H. (2021). Genome chaos, information creation, and cancer emergence: Searching for new frameworks on the 50th anniversary of the "war on cancer. Genes 13 (1), 101. 10.3390/genes13010101 35052441 PMC8774498

[B5] Iourov I. Y. Vorsanova S. G. Kurinnaia O. S. Zelenova M. A. Vasin K. S. Yurov Y. B. (2021). Causes and consequences of genome instability in psychiatric and neurodegenerative diseases. Mol. Biol. 55 (1), 42–53. 10.31857/S0026898421010158 33566024

[B6] Iourov I. Y. Vorsanova S. G. Yurov Y. B. Kutsev S. I. (2019). Ontogenetic and pathogenetic views on somatic chromosomal mosaicism. Genes 10 (5), 379. 10.3390/genes10050379 PMC6562967 31109140

[B7] Iourov I. Y. Vorsanova S. G. Yurov Y. B. (2012). Single cell genomics of the brain: Focus on neuronal diversity and neuropsychiatric diseases. Curr. Genomics 13 (6), 477–488. 10.2174/138920212802510439 23449087 PMC3426782

[B8] Iourov I. Y. Vorsanova S. G. Yurov Y. B. Zelenova M. A. Kurinnaia O. S. Vasin K. S. (2020). The cytogenomic "theory of everything": Chromohelkosis may underlie chromosomal instability and mosaicism in disease and aging. Int. J. Mol. Sci. 21 (21), 8328. 10.3390/ijms21218328 PMC7664247 33171981

[B9] Iourov I. Y. Yurov Y. B. Vorsanova S. G. Kutsev S. I. (2021). Chromosome instability, aging and brain diseases. Cells 10 (5), 1256. 10.3390/cells10051256 34069648 PMC8161106

[B10] Iourov I. Y. (2022). Svetlana G. Vorsanova (1945-2021). Mol. Cytogenet. 15 (1), 35. 10.1186/s13039-022-00613-1 35986338 PMC9390095

[B11] Miller M. B. Reed H. C. Walsh C. A. (2021). Brain somatic mutation in aging and Alzheimer's disease. Annu. Rev. Genomics Hum. Genet. 22, 239–256. 10.1146/annurev-genom-121520-081242 33979534 PMC8612367

[B12] Vijg J. (2014). Somatic mutations, genome mosaicism, cancer and aging. Curr. Opin. Genet. Dev. 26, 141–149. 10.1016/j.gde.2014.04.002 25282114 PMC5480293

[B13] Vorsanova S. G. Yurov Y. B. Iourov I. Y. (2020). Dynamic nature of somatic chromosomal mosaicism, genetic-environmental interactions and therapeutic opportunities in disease and aging. Mol. Cytogenet. 13, 16. 10.1186/s13039-020-00488-0 32411302 PMC7206664

[B14] Ye C. J. Sharpe Z. Heng H. H. (2020). Origins and consequences of chromosomal instability: From cellular adaptation to genome chaos-mediated system survival. Genes (Basel) 11 (10), 1162. 10.3390/genes11101162 PMC7601827 33008067

[B15] Ye C. J. Stilgenbauer L. Moy A. Liu G. Heng H. H. (2019). What is karyotype coding and why is genomic topology important for cancer and evolution? Front. Genet. 10, 1082. 10.3389/fgene.2019.01082 31737054 PMC6838208

[B16] Yurov Y. B. Vorsanova S. G. Iourov I. Y. (2019). Chromosome instability in the neurodegenerating brain. Front. Genet. 10, 892. 10.3389/fgene.2019.00892 31616475 PMC6764389

